# Investigation of mRNA expression levels of DNA damage response genes in Merkel Cell Polyomavirus-positive Merkel Cell Carcinoma: a pilot study

**DOI:** 10.1007/s12672-025-02651-8

**Published:** 2025-05-21

**Authors:** Sara Passerini, Matteo Fracella, Amedeo Ferlosio, Ugo Moens, Carolina Scagnolari, Guido Antonelli, Marco Ciotti, Valeria Pietropaolo

**Affiliations:** 1 Department of Public Health and Infectious Diseases, Sapienza University of Rome, 00185 Rome, Italy; 2 Department of Molecular Medicine, Laboratory of Virology, Sapienza University of Rome, Rome, Italy; 3 Anatomic Pathology, Department of Biomedicine and Prevention, Tor Vergata University of Rome, Rome, Italy; 4 Department of Medical Biology, Faculty of Health Sciences, University of Tromsø‐The Arctic University of Norway, Tromsø, Norway; 5 Virology Unit, Polyclinic Tor Vergata Foundation, Rome, Italy

**Keywords:** MCPyV, MCC, DDR, LT, *ATM*, *ATR*

## Abstract

Merkel Cell Polyomavirus (MCPyV) is recognized as the major aetiological agent of Merkel Cell Carcinoma (MCC), an aggressive skin tumor. MCPyV-mediated oncogenesis is strictly dependent on viral integration and the expression of a truncated form of the Large T Antigen (LT). Moreover, like other oncogenic DNA viruses, MCPyV may interfere with the DNA damage response (DDR) machinery, thus promoting genomic instability and tumorigenesis. Therefore, the objective of this study was to characterize MCPyV infection in 7 MCC patients and to elucidate the plausible role of the virus in the DDR pathway. MCPyV DNA was detected in 3/7 MCC patients and, as expected, viral integration and LT truncation were observed in virus-positive MCCs, along with the expression of early genes only. Over-expression of DDR genes such as *ATM*, *ATR* and their downstream kinases *Chk1* and *Chk2* was reported in MCPyV-positive MCCs supporting the potential role of the virus in interfering with DDR. Our findings support the established viral aetiology of MCC, and describe, for the first time, an over-expression of DDR components in MCPyV-positive MCC, laying the basis for future studies aimed at investigating the contribution of this pathway to MCPyV-mediated carcinogenesis and exploring the plausible clinical implications of host DDR factors for the treatment of MCC.

## Introduction

Merkel Cell Carcinoma (MCC) is a highly aggressive form of skin cancer with high rates of metastasis and mortality [1]. In addition to ultraviolet (UV)-induced DNA damage in MCC development, Merkel Cell Polyomavirus (MCPyV) has been implicated in the pathogenesis of MCC [2]. Like other Human Polyomaviruses (HPyVs), MCPyV has a small circular double-stranded DNA (dsDNA) genome that is functionally divided into early and late coding regions separated by a Non-Coding Control region (NCCR). The early region encodes the Large T (LT), small T (sT) and 57 kT antigens as well as an alternate LT open reading frame (ALTO) product [3]. The late region, instead, encodes for the major capsid protein, Viral Protein 1 (VP1) and the minor capsid protein VP2 and for two mature microRNAs (miRNAs), referred to as mcv-miR-M1-5p and mcv-miR-M1-3p which are thought to regulate the viral cycle by modulating early genes’ expression [4, 5]. In MCPyV-positive MCC, the viral genome is clonally integrated; moreover, a wild-type sT and a truncated form of LT (tLT), which eliminates the ability of the integrated virus to replicate, are constitutively expressed [2, 4]. Specifically, mutations in the carboxy-terminus of LT result in this truncated form of the protein, which lacks the helicase activity required for viral replication but retains oncogenic properties, such as the capability to bind and inactivate the retinoblastoma protein (pRb) [6, 7]. To date, most studies have focused on the oncogenic properties of LT; however, sTAg has also been implicated in cell transformation, although its mechanism of action in the development and progression of MCC remains to be further clarified [8]. Emerging evidence suggests that a variety of oncogenic DNA and RNA viruses target the host DNA damage response (DDR) [9]. The host DDR system is a complex network of proteins that collectively recognize DNA damage and coordinate cell cycle regulation with DNA repair [10]. The two key players in this signaling pathway are ataxia telangiectasia mutated (ATM) and ATM- and Rad3-related (ATR) kinases. The ATM kinase pathway is primarily activated by dsDNA breaks (DSBs), whereas ATR primarily responds to single-stranded breaks (SSBs). Activated ATR and ATM phosphorylate the downstream kinases checkpoint kinase 1 (Chk1) and checkpoint kinase 2 (Chk2), respectively [11]. So far, several groups have shown that polyomaviruses (PyVs) use the DDR machinery to replicate. In particular, previous studies have reported that DDR activation prolongs S-phase by preventing progression to mitosis, which extends the opportunity for viral DNA replication [12]. Focusing on MCPyV, it has been demonstrated that viral infection activates both the ATM and ATR arms of DDR, while MCPyV LT expression alone predominantly activates ATR, suggesting that MCPyV oncoproteins act synergistically to drive carcinogenesis [13]. In addition, several DDR proteins associated with the ATM and ATR pathways were shown to co-localize with MCPyV LT in MCPyV-infected cells [14]. However, the complex relationship between MCPyV and DDR remains to be defined. Based on this background, the current study aimed to characterize MCPyV infection in MCC tumors by investigating viral DNA, transcripts, miRNAs, as well as integration sites and LT truncation, and to examine cellular miR-375, which is known to be over-expressed in virus-positive (VP) tumors [15]. In addition, the study focused on whether MCPyV infection affects the expression of DDR genes such as *ATM*, *ATR*, *Chk1* and *Chk2* in MCC tumors.

## Methods

**Fig. 1:**
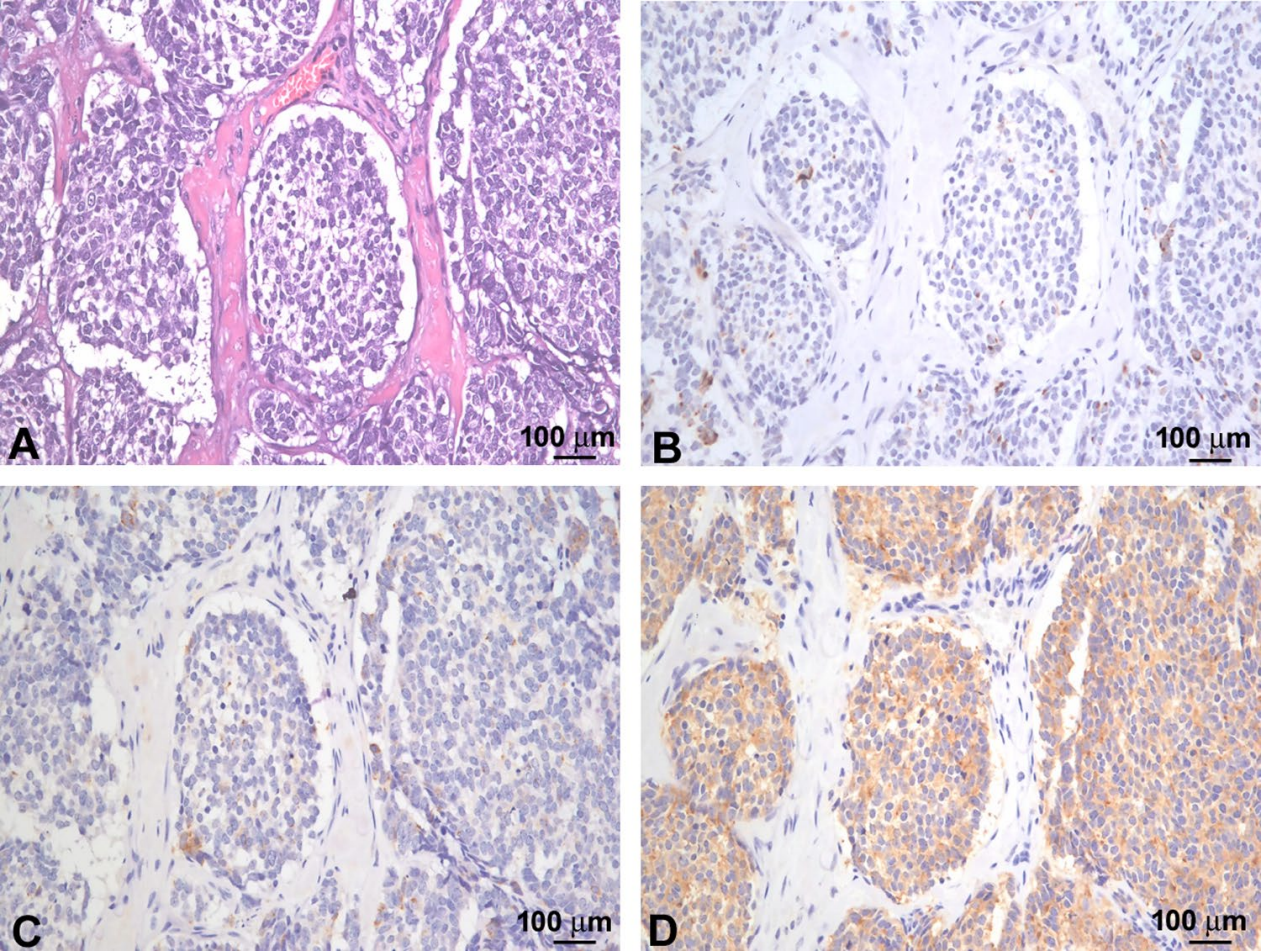
Microscopic and immunophenotypic features of a case of MCC. **A** Sub-epidermal neoplastic nodule characterized by small-intermediate cells with “salt and pepper” chromatin and scant cytoplasm resulted positive for CK20 (**B**), chromogranin A (**C**) and synaptophysin (**D**) (original magnification 200×)

Formalin-fixed paraffin-embedded (FFPE) tissues were obtained from 7 MCC patients (7 males, age range 48–93 years, mean age ± standard dev. 72 ± 12.85 years) at the Dermatology Clinic of Tor Vergata University Hospital (Rome, Italy). The study was carried out according to the Declaration of Helsinki, and approval was granted by the institutional review board and the Ethics Committee of the University Hospital Tor Vergata (Rome, Italy), under protocol number 0015440/2019, 1 July 2019. The diagnosis of MCC was suspected when a microscopic sample showed an expansive, nodular or diffusely infiltrating tumor within the dermis or subcutis characterized by a small round blue cell tumor with high nucleus: cytoplasm ratio, finely dispersed chromatin (salt and pepper), indistinct nucleoli and scant cytoplasm. Conspicuous mitoses and apoptotic bodies with variable nuclear molding and crush artefacts were frequently observed. According to international diagnostic criteria, MCC typically express neuroendocrine markers (chromogranin A and synaptophysin) and characteristic peri-nuclear dot-like positivity of CK20, CAM5.2 and pan cytokeratins while CK7, TTF1, LCA, S100 and cdx2 are negative (Fig. 1).

For the biomolecular study, FFPE tissues without necrosis were selected, with a percentage of tumoral area > 50% and more than 5000 tumoral cells counted by a pathologist. After total DNA extraction using the Quick-DNA FFPE kit (Zymo Research, Irvine, CA, USA), MCPyV DNA was detected by quantitative polymerase chain reaction (qPCR), using primers and probe for MCPyV *sTAg*, as previously described [16, 17]. Viral load, expressed as copies/milliliter (copies/mL), was determined using standard curves from tenfold plasmid dilutions (pMCV-R17a, Addgene, #24729), with a detection limit of 10 DNA copies/reaction. Samples positive for MCPyV DNA were further analyzed by standard PCR using primers for the LT (LT1 and LT3), NCCR and VP1 regions [18, 19]. Moreover, the amplified products were purified by the miPCR purification kit (Metabion, Planneg, Germany) and sequenced (Bio-Fab research, Rome, Italy), to examine NCCR and VP1 variations. Alignment between the obtained sequences and the reference strain (GenBank strain: MCC350, EU375803) was then performed using the Clustal W2 program. Viral integration sites were investigated by the detection of the integrated papilloma sequence (DIPS)–PCR technique [20, 21] and full-length* LT* sequencing was performed using a combination of six different primer sets designed to amplify DNA sequences from nucleotide position 151 to 31,102 (GenBank strain EU375803), corresponding to the entire MCPyV *LT* [21]. In parallel, total RNA, including miRNA, was extracted with the FFPE RNA purification kit (Norgen, Thorold, ON, Canada). The extracted RNA was reverse transcribed using the SensiFAST cDNA Synthesis kit (Meridian Bioscience, Cincinnati, OH, USA) and used to investigate MCPyV *LT* and *VP1* gene expression by PCR [21, 22]. Pre-designed TaqMan microRNA assays (Thermo Fisher Scientific, Waltham, MA, USA) for mcv-miR-M1-5p (ID 006356) and miR-375 (ID000564) were used to measure relative levels of miRNAs expression. RNU6B (ID001093) was used as an endogenous control to normalize miRNA expression. Relative miRNAs expression levels were calculated by the comparative ΔCt method and reported as 2 ^− ΔCt^. Quantitative analysis of mRNA expression values of *ATM*, *ATR*, *Chk1* and *Chk2* was performed by RT-qPCR employing previously described primers [22, 23]. β-globin was used as a housekeeping gene to normalize the obtained mRNA levels. Therefore, the relative expression of the genes was expressed as 2 ^− ΔCt^. For statistical analysis, gene expression values were tested using the Shapiro–Wilk test to assess normal distribution. Mean values of miR-375 and DDR genes in MCPyV-positive and negative groups were compared by independent samples T-test. *p* < 0.05 were considered statistically significant. Statistical analysis was performed using SPSS (version 28.0).

## Results

### MCPyV detection in MCC tumors

**Fig. 2:**
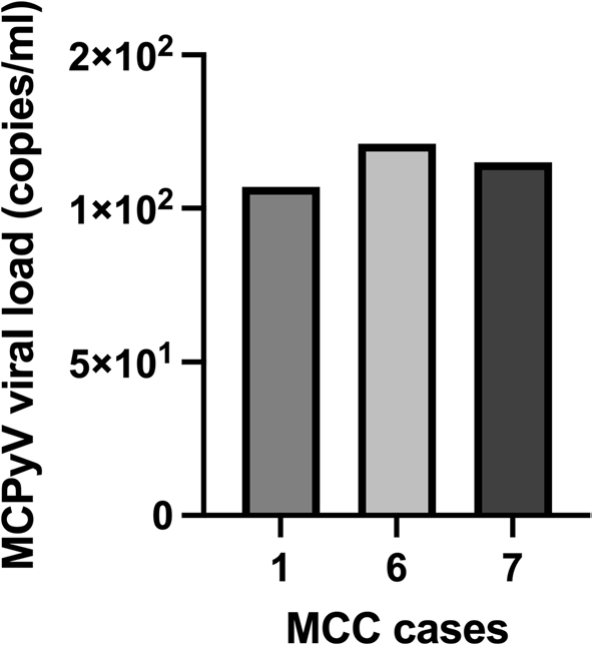
MCPyV viral loads in MCC cases

In total, 3/7 (42.8%) MCC samples were tested positive for MCPyV sT DNA with viral loads of 1.07 × 10^2^, 1.21 × 10^2^ and 1.15 × 10^2^ copies/ml, respectively (Fig. 2), and a mean viral load of 1.14 × 10^2^ copies/ml. The same samples were further positive for LT1 and LT3 amplification and for MCPyV NCCR and VP1 regions (Table 1).

**Table 1:** MCPyV viral load and molecular state in MCPyV positive MCC

Case n°	MCPyV DNA load (copies/ml)	LT		NCCR	VP1	LT	LT RNA	VP1 RNA	Integration sites	
		LT1	LT3						Viral junction	Cellular junction
1	1.07 × 10^2^	+	+	Canonical	Canonical	Truncated	+	–	2597 (LT)-3′	5q11.2
6	1.21 × 10^2^	+	+	Canonical	Canonical	Truncated	+	–	1399 (LT)-3′	5q11.2
7	1.15 × 10^2^	+	+	Canonical	Canonical	Truncated	+	–	2843(LT)-3′	5q11.2

### Viral sequences analysis

Sequencing analysis of amplified NCCR revealed canonical sequences in all samples analyzed. The same was observed for the VP1 region, which showed a high degree of homology with the reference strain MCC350. In addition, LT sequencing reported frameshift mutations that generated stop codons, eliminating the LT helicase domain but leaving the Rb-binding domain intact (Table 1).

### Integration of MCPyV genome

Viral integration sites were identified in all VP-tumors where MCPyV DNA was inserted into the long arm of chromosome 5 and viral host junctions were located at nucleotide positions 2597, 1399 and 2843 of the MCPyV *LT* gene (Table 1).

### Expression of LTAg and VP1 transcripts

In MCPyV-positive MCC, only *LT* gene expression was reported, whereas *VP1* sequence was not detected at the RNA level (Table 1).

### miRNA expression

**Fig. 3:**
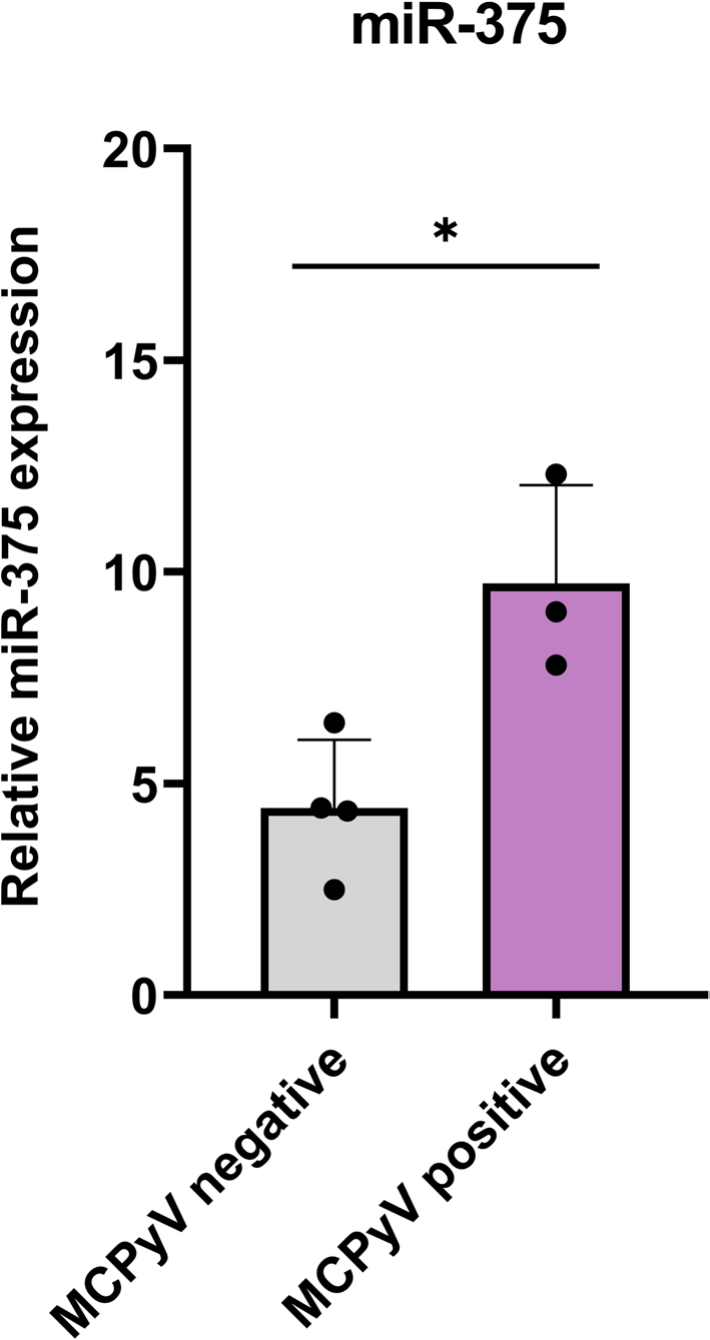
miR-375 relative expression in MCPyV-negative and MCPyV-positive MCCs. Mean values of miR-375 in MCPyV positive and negative groups were compared by independent samples T-test. *p < 0.05; **p < 0.01; ***p ≤ 0.001

MCC specimens were also examined for MCPyV-encoded miR-5p and cellular miR-375. qPCR results showed that mcv-miR-M1-5p was undetectable in all analyzed samples. miR-375 expression values followed a normal distribution (Shapiro–Wilk test; *p* > *0.05*). A significant over-expression was reported in virus-positive tumors compared to negative ones (*p* = 0.016) (Fig. 3).

### DDR genes expression levels

**Fig. 4:**
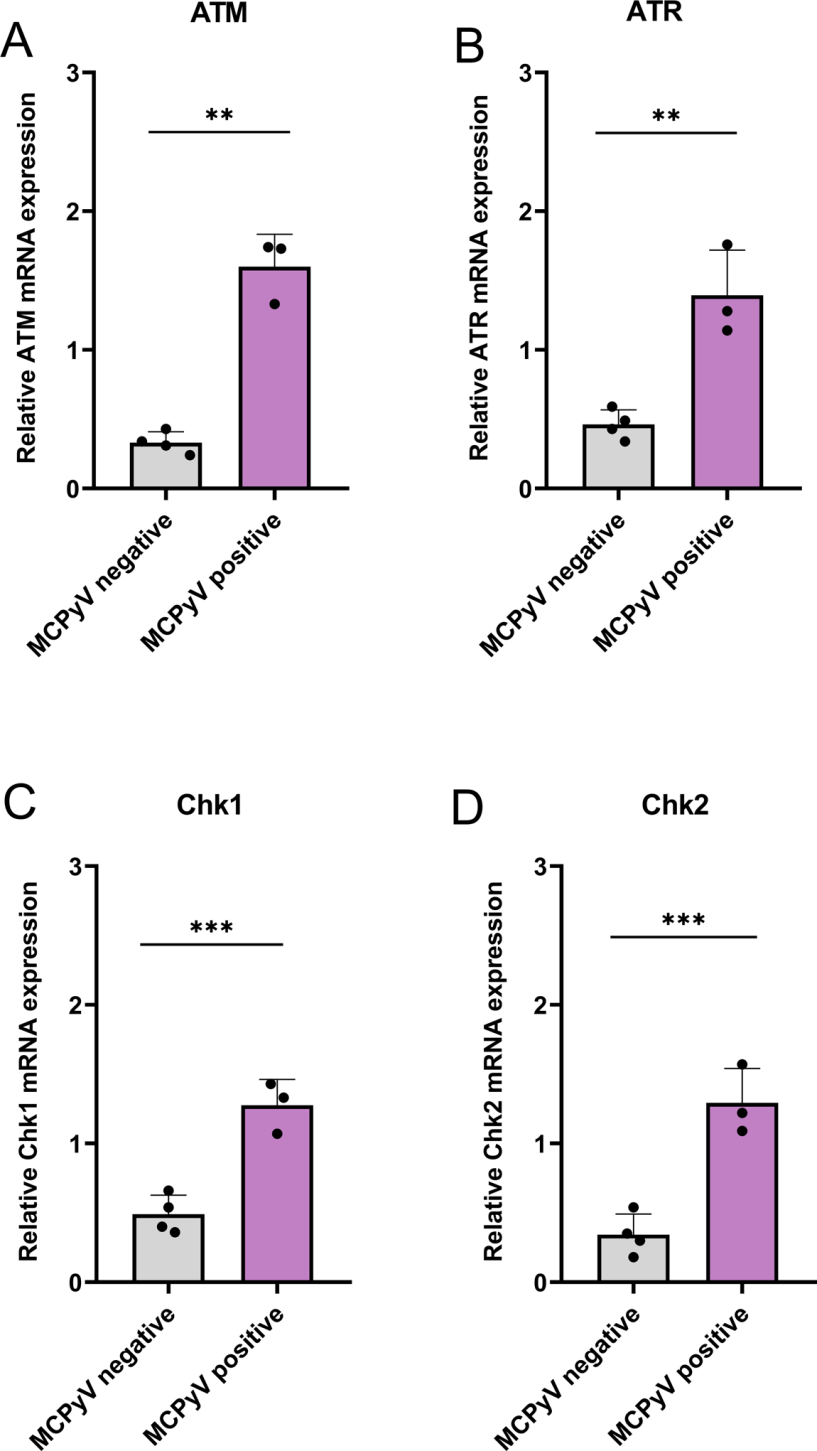
Relative expression of DDR genes mRNAs in MCPyV-negative and MCPyV-positive MCCs. Mean values of DDR genes (ATM, Panel **A**; ATR, Panel **B**; Chk1, Panel **C**; Chk2, Panel **D**) in MCPyV-positive and negative groups were compared by independent samples T-test. *p < 0.05; **p < 0.01; ***p ≤ 0.001

Gene expression values followed a normal distribution (Shapiro–Wilk test; *p* > *0.05*). Analysis of DDR genes such as* ATM*, *ATR* and their downstream kinases *Chk1* and *Chk2*, showed higher expression in MCPyV-positive MCCs compared to virus-negative ones (Fig. 4; ATM, Panel A,* p* = 0.007; ATR, Panel B, *p* = 0.003; Chk1, Panel C, *p* = 0.001; Chk2, Panel D, *p* = 0.001).

## Discussion

It is now recognized that oncogenic viruses exploit cellular pathways to replicate their genome. Specifically, some viruses are able to manipulate the DDR pathway, a complex signaling mechanism that detects and repairs DNA damage to maintain the integrity of the cellular genome [24]. Several studies have reported that Human Papillomavirus (HPV) recruits the DDR machinery to favor viral DNA replication and expression of viral oncogenic proteins [25]. In view of the similarities described between HPV and MCPyV, a plausible role for this virus in the manipulation of the DDR pathway has been discussed. Notably, MCPyV is known to be the major causative agent of MCC; therefore, investigating MCPyV biology and its contribution to the DDR pathway in MCC may help to elucidate viral pathogenesis. However, the difficulty in obtaining and analyzing MCC tissue poses a major challenge. Of the MCC samples analyzed, 3 were tested positive for MCPyV, reinforcing the well-established viral etiology of this type of tumor. Consistent with previous studies, no significant mutations were observed in NCCR and VP1 DNA sequences, supporting that variations are not associated with carcinogenesis [3, 21]. Moreover, as expected, only early genes were detected at the RNA level, supporting the suppression of viral replication is essential for MCPyV-mediated oncogenesis [18, 21]. In addition, viral integration and LT truncation were observed, strongly confirming their crucial role in MCPyV-mediated transformation [2]. MCPyV-encoded miRNAs were not detectable, so their function in virus-induced MCC remains unclear. However, when cellular miR-375 was analyzed, a significant over-expression was observed in MCPyV-positive MCC, contributing to the possibility of considering this miRNA as a *biomarker* for VP-MCC [15]. In addition to the characterization of MCPyV infection in the pathogenesis of MCC, a key aim of this study was also the elucidation of the impact of MCPyV infection on the DDR pathway. Previous studies have described a link between DDR molecules and MCPyV T antigens. More specifically, in vitro experiments emphasized the active role of MCPyV sT and LT in activating the DDR pathway [13, 14, 26]. In the current studies, we examined the expression of the *ATR* and *ATM* genes and their respective downstream molecules, *Chk1* and *Chk2,* at the RNA level. Our results showed a higher expression of these mRNAs in VP-tumors compared to negative ones, supporting previous data indicating the capability of MCPyV antigens to activate the DDR pathway at multiple levels [13, 14]. Overstimulation of both branches of DDR was observed in tumors expressing tLT, supporting recent findings about the centrality of tLT-dependent DNA damage control in MCC [27]. However, previous studies reported that MCPyV activates the ATR/Chk1 pathway through its LT C-terminal region [13]. Therefore, it may be speculated that additional components in viral infection may contribute to DDR activation. Moreover, since episomal and integrated viral DNA copies can coexist in the same MCC sample, the episomal MCPyV may reinforce ATM and ATR activation by expressing the full-length form of LT. Based on previous results with BKPyV and JCPyV, it is controversial that ATM and ATR may be involved in MCPyV infection by affecting cell cycle status [28]. As observed in JCPyV-infected cells, where activation of ATM and ATR leads to G2 arrest contributing to viral replication by maintaining the cellular replication machinery and preventing mitosis [29], MCPyV infection may induce DDR pathway activation, thereby favoring its DNA replication. This hypothesis is also supported by in vitro results showing that MCPyV LT-induced ATR activation leads to a modest G2 arrest, thus creating a cellular environment favorable to viral DNA replication [13]. Despite the increasing evidence for DDR activation upon viral infection, how this activation is implicated in MCPyV replication and mediated oncogenesis remains to be clarified. As previously proposed, ATR- and ATM-mediated DDR may cause an abnormal MCPyV DNA replication leading to viral integration and oncogenic progression; on the other hand, overstimulation of host DDR may promote viral DNA replication by repairing chromosomal damage caused by MCPyV infection on its DNA [14]. This latter hypothesis would be desirable in the context of MCC tumors, as DDR activation could be an important therapeutic target in the future. While our findings suggest a potential link between MCPyV infection and the activation of the DDR in MCC tumors, here we provide insights that need to be further validated due to certain limitations. Notably, the small number of samples available for analysis highlights the need for further research in a larger cohort of patients to increase the statistical power and reliability of our findings. Expanding the sample size will be essential to validate our results and strengthen the significance of this pilot study in a broader context, especially for diagnostic and therapeutic purposes. Additionally, our analysis relies on mRNA, which, while providing sensitive data about the over-expression of DDR genes, does not directly confirm DDR activation at the protein level. Therefore, further studies aimed at investigating DDR proteins and their post-translational modification will contribute to delineate the relevance of DDR in virus-associated tumors.

In conclusion, our results provide new insight into the capability of the virus to trigger DDR pathway activity in virus-positive MCC, starting with transcriptional activation of DDR kinase genes. Based on these findings, further research is needed to fully elucidate the downstream effect of this transcriptional activation, thus contributing to better delineating the relevance of this pathway in virus-mediated carcinogenesis and to explore the plausible clinical implications of host DDR factors for MCC treatment. Specifically, future experiments should characterize the DDR pathway in MCC cell lines following MCPyV infection to evaluate the mechanistic role of DDR in the MCPyV life cycle.

## Data Availability

The data that support the findings of this study are available on request from the corresponding author. The data are not publicly available due to privacy or ethical restrictions.
